# Maternal Hyperandrogenemia and the Long-Term Neuropsychological, Sex Developmental, and Metabolic Effects on Offspring

**DOI:** 10.3390/ijms26052199

**Published:** 2025-02-28

**Authors:** Menelaos Darlas, Sophia Kalantaridou, Georgios Valsamakis

**Affiliations:** 1 1st Department of Obstetrics and Gynecology, ‘Alexandra’ General Hospital, National and Kapodistrian University of Athens, 80 Vasilissis Sofias Avenue, 11528 Athens, Greece; mdarlas2110@gmail.com; 2 3nd Department of Obstetrics and Gynecology, ‘Attikon’ General Hospital, National and Kapodistrian University of Athens, 11528 Athens, Greece; sophiakalantaridou@gmail.com; 3 2nd Department of Obstetrics and Gynaecology, Aretaieio Hospital, School of Medicine, National and Kapodistrian University of Athens, 11528 Athens, Greece

**Keywords:** hyperandrogenemia, pregnancy, sex ratio, sex developmental, offspring

## Abstract

Maternal hormonal and metabolic disorders, such as diabetes and obesity, can adversely affect the intrauterine environment, resulting in suboptimal fetal growth and an elevated risk of cardiovascular and metabolic diseases in the later life of the offspring. In this review, we examine the long-term impact of elevated maternal androgen levels during pregnancy on offspring. Maternal hyperandrogenemia is linked to various neurodevelopmental disorders, including attention-deficit/hyperactivity disorder, autism spectrum disorder, and anxiety-like behaviors, mediated by alterations in key brain regions responsible for emotion and cognition. Furthermore, children born to mothers with hyperandrogenemia exhibit heightened risk of metabolic and cardiovascular dysfunctions, such as obesity, insulin resistance, and hypertension, which can manifest early in life. Prenatal exposure to androgens has also been linked to reduced birth weights and altered fetal growth, potentially due to impaired placental function. Additionally, maternal testosterone levels influence offspring sex ratios, often favoring male births, though exceptions occur in certain conditions, such as congenital adrenal hyperplasia. The findings of this review underscore the need for healthcare professionals to monitor maternal serum androgen profiles during pregnancy. Further research is needed to determine underlying mechanisms and potential interventions to mitigate these risks.

## 1. Introduction

The intrauterine environment is crucial for the growth and development of the fetus in utero. Intrauterine conditions are determined not only by placental function but also by maternal pathology. For example, maternal hormonal conditions such as diabetes and obesity can alter the intrauterine environment, leading to inadequate fetal growth and an increased risk of cardiovascular and metabolic diseases in the later life of the offspring [1,2,3]. Thyroid hormones and glucocorticoids are also known to regulate fetal development and have long-term effects on offspring [4,5].

**Figure 1:**
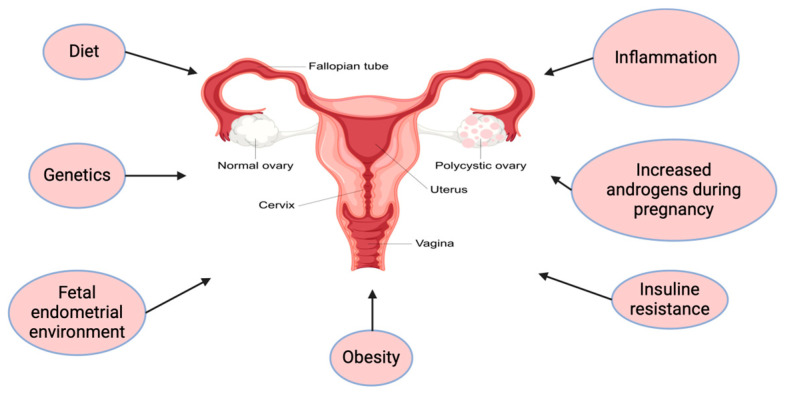
Causes of PCOS. This figure illustrates the primary factors that can exacerbate PCOS, including inflammation, insulin resistance, increased androgen levels during pregnancy, genetic predisposition, diet, obesity, and fetal endometrial environment. These interconnected factors contribute to the hormonal imbalance and metabolic dysregulation observed in PCOS, highlighting its multifactorial etiology.

Androgens are essential for the physiological regulation of pregnancy in females. The circulating concentrations of androgens (testosterone, dihydrotestosterone, androstenedione, and dehydroepiandrosterone) show significant changes during the normal progression of pregnancy [6]. Testosterone increase plays a role in supporting pregnancy maintenance and initiating the labor; in comparison, dihydrotestosterone and dehydroepiandrosterone (DHEAS) promote cervical ripening and contribute the relaxation of the myometrium according to studies carried out in humans [7,8]. However, high levels of circulating male sex hormones in pregnancy can be the result of a number of diseases/conditions and can be associated with detrimental consequences for the fetus [7]. Prenatal exposure to high concentrations of androgens (either of fetal or maternal origin) could be a source of prenatal programming with consequences during adulthood. The most common causes of androgen excess in women include polycystic ovary syndrome, congenital adrenal hyperplasia, and adrenal tumors. During pregnancy, luteoma and placental aromatase deficiency are additional causes of hyperandrogenism [8]. Polycystic ovary syndrome (PCOS), defined by oligoovulation or anovulation, clinical and biochemical androgen excess, and polycystic ovarian morphology, is responsible for 70% of cases of hyperandrogenemia [9]. Therefore, it is the most common cause of hyperandrogenemia during pregnancy [10]. The etiology of PCOS is complex and multicausal, including a combination of genetic, metabolic, and environmental factors [11]. The main mechanism involved in the pathogenesis of PCOS is thought to be insulin resistance [12]. Insulin, through its receptor, controls ovarian steroidogenesis and disrupts follicular development [13]. Insulin also indirectly influences the prevalence of hyperandrogenism by decreasing the liver’s production of sex hormone-binding globulin (SHBG), thus increasing serum androgen levels [13]. Genetic predispositions and low-grade inflammation lead to further androgen excess [14]. PCOS is also linked to metabolic complications, including obesity, insulin resistance, type 2 diabetes, and dyslipidemia, thus exacerbating its effects on pregnancy [15,16] (Figure 1). A large body of published data links prenatal androgen excess and adverse pregnancy outcomes [17]. The results of several studies highlight the fact that increased maternal levels of testosterone in the third trimester are linked to pre-eclampsia; gestational diabetes; preterm delivery; and developmental, metabolic, and reproductive alterations in offspring, suggesting testosterone as a predictive marker [17,18]. Moreover, high levels of testosterone and a high testosterone-to-sex hormone-binding globulin (T/SHBG) ratio have been observed in women with recurrent miscarriages [18].

However, there is a lack of literature regarding the effects of maternal hyperandrogenemia during pregnancy on offspring. In this review, we aim to explore the long-term neuropsychological, sexual developmental, and metabolic effects of maternal androgen excess during pregnancy on offspring.

## 2. Effects on the Neuropsychiatric System of the Offspring

### 2.1. Background

**Figure 2:**
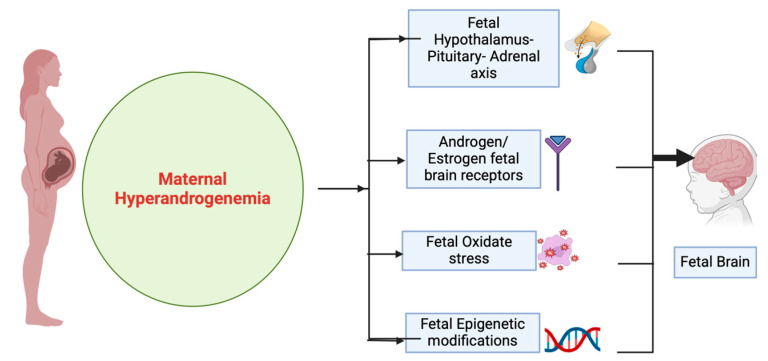
Molecular mechanisms of the effects of maternal hyperandrogenemia in the fetal brain. The above figure illustrates the molecular pathways through which maternal hyperandrogenemia influences fetal brain development. Key mechanisms include altered hormonal signaling, epigenetic modifications, oxidative stress in the fetal environment, and changes in neuronal connectivity. These effects may contribute to long-term neurodevelopmental and behavioral outcomes in offspring.

Autism spectrum disorder (ASD), attention-deficit/hyperactivity disorder (ADHD), anxiety, and chronic tic disorder (CTD) are often not diagnosed in children due to differences in signs and symptoms [19]. The prevalence of these behavioral disorders varies from 0.1% to 24% in children and youth, and their etiology is not entirely understood [20,21]. Environmental, genetic, and environment–gene interaction factors can lead to adverse neurodevelopmental outcomes [22]. Thus, it is crucial to identify the risk factors for neuropsychiatric disorders in children in order to facilitate earlier screening and management in the pediatric population. There is strong evidence indicating that prenatal androgen exposure may play a causal role in the development of neuropsychiatric disorders in children [23]. Maternal androgens influence fetal brain development and function through several molecular mechanisms. Elevated maternal androgen levels can influence the expression of androgen and estrogen receptors in the fetal brain, impacting the development of crucial regions such as the hypothalamus, amygdala, and prefrontal cortex [24]. Additionally, elevated maternal androgen levels may interact with the hypothalamic–pituitary–adrenal (HPA) axis, altering fetal cortisol levels, which can influence neural circuits related to stress responses [25]. Androgens can also indirectly affect thyroid hormone pathways, which are critical for neurodevelopment [26]. Furthermore, maternal androgens may act through epigenetic modifications, such as DNA methylation and histone acetylation, influencing gene expression patterns that are critical for neural differentiation and connectivity [27]. In addition, excess androgens may increase oxidative stress in the fetal environment, disrupting neural differentiation and maturation [28] (Figure 2). In addition, anti-androgen treatment shows a response in patients affected by certain psychiatric disorders, such as TD/CTD [29]. Additionally, studies have shown that maternal testosterone affects brain morphology and function and correlates with neural development and mental function [30]. The most distinctive feature of PCOS is high levels of androgens, and women with PCOS during pregnancy exhibit increased circulating androgen levels. Moreover, the placentas of women with PCOS exhibit abnormal steroidogenesis and enhanced capacity for producing androgens [31]. Many researchers have therefore utilized PCOS as a model of maternal hyperandrogenemia.

### 2.2. Attention-Deficit/Hyperactivity Disorder

ADHD is among the most prevalent neurodevelopmental disorders. Based on data from WHO World Mental Health Surveys, the prevalence of ADHD averages 2.8% [32]. According to DSM-IV, the disorder is characterized by pervasive symptoms of ADHD and is categorized into three types: inattentive, hyperactive–impulse, or both inattention and hyperactive–impulsive symptoms. The causes of ADHD are unknown in the majority of cases; however, there are a number of genetic and environmental factors that contribute to the development of ADHD. Prenatal androgen exposure affects the neurodevelopment of offspring and increases the prevalence of ADHD through various mechanisms. Firstly, brain regions such as the reward system, motor function, and spatial memory are influenced by sex hormones [33]. Animal studies have shown that sex hormone exposure is associated with abnormal activity in the dopaminergic system, which is impaired in ADHD [34]. Moreover, anomalous hemispheric asymmetries as seen in ADHD have been related to high fetal testosterone concentrations [35].

A nationwide register-based cohort study was carried out to explore the impact of prenatal androgen exposure on the development of ADHD [36]. This study used maternal PCOS as a model of offspring exposure to elevated levels of prenatal androgens, using a sample consisting of 58,912 ADHD cases matched to 499,998 controls for the period from 1984 to 2011. The results indicated that maternal diagnosis of PCOS is linked to a higher likelihood of ADHD in offspring even after excluding cases with comorbid ASD. In parallel, the results of two recent reviews [37,38] based on the findings of 19 articles and 6 articles, respectively, confirm that maternal PCOS contributes to the risk of neurodevelopmental and behavioral disorders in children and, more specifically, the development of ADHD.

According to Maliqueo [39], prenatal androgen exposure may elevate the risk of ADHD by influencing the pathways involved in fetal brain development. The findings of this study reinforce the idea that a hyperandrogenic environment affects dendritic morphology, nerve density, and abnormal synapse function and morphology. Furthermore, inflammatory cytokines, chronic inflammation, and altered gut microbiota in PCOS mothers may also affect fetal brain development [40]. Taken together, these findings indicate that high androgen exposure influences fetal brain development through various mechanisms, and, thus, hyperandrogenemia is a strong risk factor for ADHD.

### 2.3. Autism Spectrum Disorders

ASD is a condition related to brain development that impacts personal socialization and communication. It is more prevalent in boys, with a male-to-female ratio of 4:1 [41]. Given the capacity of sex hormones to affect brain areas relevant to ASD, androgens are an essential risk factor [33]. In a study conducted in Denmark by Baron and Cohen [40], the authors prospectively examined the levels of steroid hormones in fetal amniotic fluid and found that amniotic fluid steroid hormones, including testosterone and androstenedione, are elevated in those who later developed ASD. However, the source of elevated steroidogenic activity could not be identified, and the fetus, mother, placenta, or other environmental factors may all have played a role to such elevations [40]. The findings of this study provide the first evidence that individuals with ASD have high steroid hormone activity during the fetal period, in addition to providing further evidence of fetal steroid hormones as a risk factor for ASD. In addition, the authors of a large-scale, nationwide case–control study conducted in Sweden (incorporating children from 4 to 17 years old born in Sweden from 1984 to 2007) found that maternal PCOS increased the likelihood of ASD in the offspring by 59% [42]. Moreover, Rotem et al. [43], in a cohort of 437,222 children, examined the association between maternal hyperandrogenemia and progeny ASD, including all of the possible causes of hyperandrogenemia (PCOS, hyperaldosteronism, anterior pituitary hyperfunction, Cushing syndrome, and congenital adrenal hyperplasia) and conditions that could be caused by excess androgens, such as alopecia and hirsutism. The results of this study strengthen previous assumptions that PCOS and maternal conditions linked to excess androgen levels are risk factors for ASD, directly implicating them in the etiology of this disorder [43]. In conclusion, the available evidence suggests that women with high levels of androgens are at an elevated risk of having a child with ASD, an effect-size estimate derived from a large number of patients in high-quality studies.

### 2.4. Anxiety-Like Behavior

Anxiety disorders represent the most common mental disorders and affect up to 33.7% of the population at some point in their lifetime [44]. These disorders can lead to other psychological and physical conditions; however, their etiology remains unknown. Researchers recently investigated the association between maternal hyperandrogenemia and anxiety disorders in offspring. The effects of fetal testosterone on brain morphology and function are correlated with neural development and mental function [45]. In a study involving rodent models of a maternal PCOS-like phenotype induced by injections of androgens, the authors found an increase in anxiety-like behavior in female and, to a lesser extent, male offspring, potentially mediated via changes in the androgenic, serotonergic, and GABAergic pathways in the amygdala and hippocampus [30]. The results of a recent Swedish nationwide cohort study by Risal et al. [46] showed that daughters of women with PCOS have a 78% increased risk of developing anxiety disorders compared to the sons of such women. Furthermore, in the same study utilizing a PCOS-like mouse model, prenatal exposure to androgens triggered anxiety-like behavior in first-generation female offspring, which was passed down transgenerationally to third-generation female offspring. In contrast, this effect was not observed in the first, second, or third generation of male siblings, highlighting a sexually dimorphic response in behavior. These behavioral changes in the third-generation female offspring provide evidence that maternal androgens in PCOS and obesity are risk factors for the transgenerational transmission of anxiety disorders in children of women with PCOS. Moreover, in a prospective birth cohort study with 1915 mother–child dyads, Robinson et al. [47] showed a positive association between maternal PCOS and anxiety disorder diagnosis and emotional symptoms in children regardless of maternal hirsutism. Taken together, these studies indicate that maternal androgen excess induces anxiety-like behavior in female and, to a lesser degree, male offspring, an effect potentially mediated by epigenetic reprogramming.

### 2.5. Other Neuropsychiatric Disorders

Androgen exposure increases the risk of variety in neurodevelopmental and psychiatric disorders in offspring. In a recent population-based cohort study in Finland (including 1,097,753 births), children to mothers with PCOS showed increased prevalence of a broad spectrum of psychiatric disorders [48]. In particular, maternal PCOS has been shown to be associated with mood disorders, anxiety disorders, sleeping disorders, intellectual disabilities, conduct disorders, tic disorders, and emotional disorders, with no significant difference between sexes. Moreover, in the same study, Chen et al. [48] indicated that the increased risk of these disorders in offspring persisted in normal-weight mothers with PCOS, suggesting that factors beyond obesity, such as hyperandrogenemia, must play a role. However, the results of the study by Cesta et al. [23] support the causal influence of prenatal androgen exposure in the development of male-predominant neuropsychiatric disorders in offspring born to mothers with PCOS.

## 3. Effects on the Metabolic and Cardiovascular System of Offspring

In general, women with hyperandrogenemia diagnosed with PCOS have an adverse cardiometabolic risk profile, which is established in childhood. According to the Barker hypothesis, the intrauterine environment affects the health and development of the child [49]. Zuchowski et al. [50] used female Sprague Dawley rats implanted with dihydrotestosterone to determine the cardiometabolic consequences in male offspring. The results of this study revealed that the male offspring of hyperandrogenemic mothers showed elevated levels of dyslipidemia, proteinuria, and increased sensitivity to Ang II infusion compared to control offspring groups, suggesting that maternal hyperandrogenemia may increase the risk of cardiometabolic abnormalities. Administration of low-dose Ang II (50 ng/mg/min) for 20 days increased mean arterial pressure in male offspring of hyperandrogenemic female dams. Rats in this subgroup exhibited higher levels of total cholesterol and higher excretion of urinary protein compared to the controls. Moreover, in a study conducted on 74 children of women who were previously diagnosed with PCOS, these children faced an elevated risk of both cardiovascular and metabolic complications [51]. These findings are supported by an increased pulse pressure and a higher left ventricular internal diameter at 2.5 to 4 years of age, and at the age of 6 to 8 years, the children of PCOS mothers had higher carotid IMT and higher serum levels of total triglycerides and LDL-cholesterol. Specifically, the larger left ventricular internal diameter could be caused by insulin or androgens influencing fetal cardiac anatomy [52]; in comparison, at 6 to 8 years old, the increased carotid IMT and elevated levels of triglycerides and LDL-cholesterol further contribute to the long-term cardiovascular impacts on children born to women with PCOS. Furthermore, the results of the systematic review and meta-analysis of Gunning et al. [53] confirm the subtle abnormalities in cardiometabolic features in offspring from PCOS pregnancies, as they exhibited differences in birthweight and HDL-cholesterol levels compared to controls.

The authors of a recent animal study investigated the role of intrauterine androgen exposure and maternal obesity in cardiovascular health in the female offspring of women with PCOS [54]. The study demonstrates that prenatal exposure to dihydrotestosterone, independent of maternal obesity, is a risk factor for cardiac hypertrophy in adult female mice offspring. Moreover, changes related to cardiac hypertrophic remodeling are evident at an early age and can have lasting effects on offspring. Consistent with these results, a study conducted on sheep with prenatally androgenized offspring induced by maternal testosterone excess was associated with an increased expression of genes involved in insulin signaling, in addition to those related to left ventricular hypertrophy and stress, in female sheep. In addition, the results of the study by Hou et al. [55] suggest that maternal testosterone excess causes cardiac hypertrophy in adult female rats through the increased expression of Pkcδ in cardiac myocytes. All of these clinical and registry studies indicate that maternal hyperandrogenemia is a risk factor for the offspring’s cardiovascular health, and it may have long-term impacts on the health of children.

The developing fetal metabolic system is acutely sensitive to the maternal hormonal environment in utero. Alterations in the concentration of hormones, such as androgen, can cause metabolic dysfunction in offspring. The findings of a cross-sectional study involving 99 daughters (30 prepubertal and 69 pubertal girls) of women with PCOS indicate the existence of metabolic abnormalities even before puberty that persist during pubertal development [56]. The results of this study suggest that exposure to maternal PCOS was associated with increased ovarian volume and 2 h insulin in daughters at all Tanner stages, and testosterone, post-stimulated LH, and 17-hydroxyprogesterone concentrations were higher in daughters at Tanner stages IV and V. Furthermore, the authors of a large, nationwide cohort study of 1,097,753 births found that maternal PCOS or anovulatory infertility increases the risk of obesity in offspring independent of sex and fertility treatment, with it being associated with diabetes diagnosis in female offspring only [57]. The bulk of this evidence is consistent with the observations of the animal model study of Carrasco et al. [58] which indicate that prenatal exposure to testosterone reduces peripheral insulin sensitivity. In this study, hyperandrogenemia in sheep mothers lowered insulin sensitivity in female offspring compared to sheep born to mothers with normal levels of androgens. Insulin sensitivity was also lower in the first group of offspring after acute administration of 40 mg of testosterone at 40 weeks of age. Carrasco et al. [58] concluded that prenatal testosterone exposure has adverse effects on insulin sensitivity and that levels of postnatal androgens may also reduce insulin sensitivity.

## 4. Effects on Birth Weight

Intrauterine growth restriction (IUGR) refers to a fetus that has not achieved its expected growth potential because of various etiologies that include maternal, placental, and genetic factors. Children with IUGR are at a higher risk of long-term problems, such as metabolic syndrome, type 2 diabetes, and cardiovascular diseases. Fetal growth restriction may be caused by fetal, placental, and maternal factors, and the most common examples leading to IUGR include hypoxia, maternal obesity, nutritional restriction, and fetal infections. In relating hyperandrogenic conditions to IUGR, the results of the prospective Scandinavian study by Carlsen et al. [59] showed a negative association between endogenous circulating maternal testosterone levels and the offspring’s birth size. A decrease in birth weight of 160 g was found after an increase in circulating maternal testosterone levels from the 25th to the 75th percentile at 17 weeks of gestation. These findings are supported by animal studies linking prenatal hyperandrogenism with size at birth. In sheep, excess prenatal testosterone levels in the mother caused decreased birth weight, height, and chest circumferences in newborns of both sexes [60]. Similarly, prenatal testosterone-treated rats exhibited a dose-dependent decrease in birth weight [61]. Taken together, excess maternal testosterone during pregnancy may be associated with fetal growth restriction in utero, and this association may be mediated by changes in placental differentiation or function.

## 5. Effects on Sex Ratio

The proportion of sexes at birth in different mammals depends on whether an X or a Y chromosome-bearing spermatozoon fertilizes the ovum first. However, the results of numerous studies have demonstrated that offspring sex ratios are frequently associated with factors such as the maternal body condition, maternal hormonal profile, and stress [62]. Moreover, maternal dominance, a characteristic associated with testosterone in female mammals, is one of the primary factors identified as influencing sex. The association between maternal testosterone levels and male offspring gender has been studied in various species, including voles, ibex, and bovines. The authors of these studies found that elevated serum testosterone concentrations in female voles are associated with a male-biased sex ratio [63]. Furthermore, the results of the study by Shargal et al. [64] demonstrated the association between fecal testosterone concentration in ibex and producing male-gender offspring. In addition, in their bovine study, Grant et al. [65] found that elevated levels of testosterone in the follicular fluid influence the sex ratio, with there being a higher likelihood that they produce male embryos after in vitro fertilization. In their study, Gharagozlou et al. [66] built on these earlier findings, showing that pre-conceptional testosterone levels impact the sex ratio of the offspring. More specifically, they found that mice treated with flutamide, which is an androgen receptor antagonist, showed a sex ratio of offspring skewed toward females, showing that testosterone pre-conceptionally affected the sex ratio of offspring. The results of all of these animal studies demonstrate the impact of maternal testosterone on offspring sex ratio and suggest that high levels of circulatory androgens are associated with a male-biased sex ratio in offspring. However, in the study by Hagenfeldt et al. [67], the authors reported a disproportionately high number of daughters born to mothers with congenital adrenal hyperplasia (CAH), which is a common cause of androgen excess. The offspring born to women with CAH numbered 19 girls and 6 boys, and compared to the control group, boys comprised 56% of the group of children born to the control mothers versus 25% born to the mothers diagnosed with CAH. When reviewing the literature, the reported number of children born to women with CAH thus far stands at 55 girls and 27 boys [67,68,69,70,71,72]. This unexpected sex ratio in offspring born to mothers with CAH requires further investigation.

## 6. Discussion

The current review was designed to assess the effects of maternal hyperandrogenemia on offspring (Table 1). Firstly, the findings of this review emphasize the significant impact of maternal hyperandrogenemia on neuropsychological outcomes in offspring. Elevated androgen levels during pregnancy can lead to a spectrum of neurodevelopmental disorders in children, such as ADHD and ASD [36,42,46]. Moreover, elevated fetal testosterone levels can adversely affect brain development and function, leading to anxiety-like behaviors, especially in female offspring [23]. Notably, all of these neuropsychological conditions appear to be influenced by hormonal alterations that affect critical brain regions responsible for emotional regulation and cognitive function. The findings of various studies, including population-based and cohort studies, underline a potential causal relationship between prenatal androgen exposure and these disorders.

Furthermore, the findings of this review indicate that maternal serum testosterone levels influence offspring sex ratios across various mammalian species, with higher concentrations typically associated with a male-biased ratio. Various animal model findings support the notion that elevated maternal testosterone levels correlate with a greater likelihood of male offspring [64,73]. However, exceptions exist, as observed in cases of congenital adrenal hyperplasia (CAH), where an unexpected skew toward female offspring challenges the prevailing assumptions about the role of androgens [66]. This anomaly indicates a need for further investigation into the mechanisms governing sex determination in the context of maternal health conditions.

Additionally, the review highlights the adverse metabolic and cardiovascular consequences for children born to mothers with hyperandrogenemia. Both clinical studies and animal models have shown that maternal hyperandrogenism can lead to a variety of adverse health outcomes in offspring, including increased dyslipidemia, hypertension, insulin resistance, and cardiac hypertrophy [51,55]. These effects seem to start early in life, with children of women with PCOS exhibiting subtle metabolic and cardiovascular abnormalities even in childhood. Furthermore, prenatal exposure to androgens has been linked to impaired insulin sensitivity, an increased risk of obesity, and metabolic dysfunction in offspring, particularly in female children [58].

Lastly, research indicates a concerning link between maternal hyperandrogenism and reduced fetal growth. Studies involving humans and animal models have shown that elevated serum levels of maternal testosterone negatively impact birth weight and overall fetal development [59,60]. These findings suggest that excess maternal testosterone may lead to fetal growth restriction through alterations in placental function and differentiation.

## 7. Conclusions

In conclusion, maternal hyperandrogenemia during pregnancy presents substantial risks for offspring, impacting both their immediate development and long-term health outcomes. The reviewed literature demonstrates a link between elevated maternal androgen levels and various neurodevelopmental and metabolic disorders in children (Table 2). The findings highlight the necessity for healthcare providers to consider maternal hormonal profiles during pregnancy as potential risk factors for offspring health. Future research efforts should focus on clarifying the underlying mechanisms by which maternal hyperandrogenemia affects fetal development and explore intervention strategies to mitigate these risks, ultimately aiming to improve maternal and child health outcomes. Furthermore, understanding the interplay between maternal hyperandrogenemia and fetal development may lead the way for targeted therapies and preventive measures in at-risk populations.

## Figures and Tables

**Table 1:** Effects of maternal hyperandrogenemia on offspring.

Organ System	Disorders	Mechanisms
Neurodevelopmental impacts	ADHD, ASD, anxiety-like behaviors, mood disorders, sleep disorders, intellectual disabilities, conduct disorders, tic disorders, and emotional disorders [36,39,40,42,43,46,48]	Altered brain development and hormonal imbalance in key brain regions (the amygdala and hippocampus) [30]
Metabolic issues	Obesity and insulin resistance [54,56,58]	Disruption of insulin sensitivity and hormonal effects on adiposity and metabolism [56,57]
Cardiovascular risks	Hypertension and cardiac hypertrophy [50,52,55]	Vascular remodeling and cardiac structural changes due to androgens [50,52,55]
Growth restriction	Low birth weight and intrauterine growth restriction [59,61]	Placental dysfunction and restricted nutrient and oxygen supply to the fetus [61]
Altered sex ratio	Male-biased sex ratio (exceptions in CAH cases) [62,64,65,67]	Elevated testosterone levels, influencing sperm–ovum interactions and embryo development [65,66]

Summary of the effects of maternal hyperandrogenemia on offspring, highlighting the impacted organ systems, associated disorders, and the underlying mechanisms. The data are derived from clinical studies, animal models, and mechanistic research, as referenced in the review.

**Table 2:** Studies providing evidence of the effects of maternal hyperandrogenemia on offspring.

Effects on Offspring	Disorder/Condition	Evidence/Studies
Neurodevelopmental effects	ADHD, ASD, and anxiety-like behavior	Swedish cohort studies and animal models [36,40,46]
Sex ratio impact	Male-biased sex ratio (exceptions in CAH cases)	Animal studies and clinical observations [65,67]
Metabolic effects	Obesity, insulin resistance, and dyslipidemia	Human cohort studies and rodent models [53,58]
Cardiovascular health	Increased risk of hypertension, cardiac hypertrophy, and vascular dysfunction	Studies in rats and sheep [50,52,55]
Birth weight and growth	Intrauterine growth restriction (IUGR) and low birth weight due to placental dysfunction	Scandinavian cohort and animal models [59,60]

The findings presented in this table highlight the multifaceted effects of maternal hyperandrogenemia on offspring, including neurodevelopmental, metabolic, cardiovascular, and growth-related outcomes. Evidence is drawn from diverse sources, including large cohort studies and animal studies underscoring the broad implications of altered maternal androgen levels during pregnancy. Exceptions, such as the female-biased sex ratio in CAH cases, indicate the need for further investigation into these mechanisms.

## Data Availability

No new data were created or analyzed in this study.
